# Intermittent Fasting Enhances Motor Coordination Through Myelin Preservation in Aged Mice

**DOI:** 10.1111/acel.14476

**Published:** 2025-01-08

**Authors:** Zhuang Liu, Ziyue Zhao, Hongying Du, Qingqing Zhou, Mei Li, Zhu Gui, Jinfeng Wu, Yunling Gao, Ning Zheng, Yu Zhang, Ailian Du, Hongxing Wang, Jie Wang

**Affiliations:** ^1^ Department of Neurology, Songjiang Research Institute, Shanghai Key Laboratory of Emotions and Affective Disorders Songjiang Hospital Affiliated to Shanghai Jiao Tong University School of Medicine Shanghai China; ^2^ State Key Laboratory of Magnetic Resonance and Atomic and Molecular Physics, Innovation Academy for Precision Measurement Science and Technology Chinese Academy of Sciences Wuhan China; ^3^ University of Chinese Academy of Sciences Beijing China; ^4^ Department of Food Science and Engineering, College of Light Industry and Food Engineering Nanjing Forestry University Nanjing China; ^5^ Department of Anesthesiology, Zhongnan Hospital Wuhan University Wuhan China; ^6^ Department of Anesthesiology First People Hospital of Foshan Foshan China; ^7^ Institute of Neuroscience and Brain Diseases; Xiangyang Central Hospital Affiliated Hospital of Hubei University of Arts and Science Xiangyang Hubei China; ^8^ Clinical & Technical Support, Philips Healthcare Shanghai China; ^9^ Division of Neuropsychiatry and Psychosomatics, Department of Neurology Xuanwu Hospital of Capital Medical University Beijing China

**Keywords:** aging animals, intermittent fasting, myelin basic protein, myelin‐associated glycoprotein, resting state functional MRI

## Abstract

Integrating dietary interventions have been extensively studied for their health benefits, such as Alzheimer's disease, Huntington's disease, and aging. However, it is necessary to fully understand the mechanisms of long‐term effects and practical applications of these dietary interventions for health. A 10‐week intermittent fasting (IMF) regimen was implemented on the aging animals in the current study. The variations of cerebral functions were analyzed employing a comprehensive experimental design that includes behavioral tests, neuroimaging, and ultrastructural analysis, such as resting‐state functional MRI (rsfMRI), EEG/EMG recordings, transmission electron microscopy, and immunohistochemistry. Over a 10‐week regimen, IMF significantly improved locomotor activity, motor coordination, and muscle strength compared to controls (*p* < 0.01). Resting‐state fMRI (rsfMRI) demonstrated that IMF modulates brain‐wide functional connectivity, enhancing communication between key brain regions. Advanced imaging techniques revealed increased expression of myelin‐related proteins, including myelin basic protein (MBP), and myelin‐associated glycoprotein (MAG), indicating enhanced myelin integrity and repair, particularly in axons with diameters < 400 nm (*p* < 0.01). These findings suggest that IMF may mitigate age‐related declines by promoting better neuronal signaling. This study highlights the potential function of IMF as a non‐pharmacological intervention to promote brain health and mitigate cognitive decline in aging populations.

## Graphical abstract

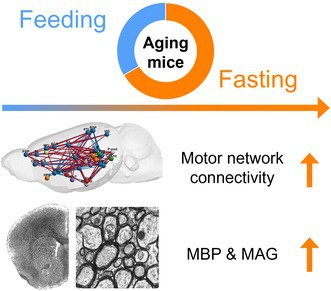

## 1 Introduction

Integrating dietary interventions such as intermittent fasting (IMF), caloric restriction (CR), or fasting mimicking diets (FMD) have been extensively studied for their health benefits, particularly concerning Alzheimer's disease, Huntington's disease, and aging (Wang et al. 2018; Whittaker et al. 2023). Aligning feeding and fasting cycles with circadian rhythms is crucial, as it synchronizes metabolic and behavioral functions, even without a central circadian clock (Longo and Panda 2016). Research indicated that IMF could improve metabolic health, promote cellular repair, reduce inflammation, and enhance longevity (De Cabo and Mattson 2019). Specifically, IMF helps regulate gut microbiota, supports intestinal regeneration, and alleviates inflammation in inflammatory bowel disease (IBD) (Rangan et al. 2019). The benefits of IMF also extend to cardiovascular health, improving cardiometabolic risk factors and endothelial function. Additionally, IMF has been associated with reduced cancer risks and enhanced cognitive functions by modulating metabolic and cellular processes (Wei et al. 2017).

Furthermore, IMF has been shown to promote beta‐cell regeneration, reverse diabetes, and decrease inflammation and oxidative stress, which are critical factors in the aging process (Wei et al. 2017; Rastogi et al. 2024). Recent studies have shown that FMD cycles also can improve gut health, reduce inflammation, and promote systemic regeneration, thereby enhancing cognitive performance and extending lifespan (Rangan et al. 2019). These findings suggest that IMF or FMD could offer significant therapeutic potential by modulating key biological pathways and promoting systemic regeneration. Despite these positive findings, further research is necessary to fully understand the mechanisms of the long‐term effects and practical applications of these dietary interventions for health and longevity.

Resting‐state functional magnetic resonance imaging (rsfMRI) has emerged as a powerful tool for exploring the brain's function (Zerbi et al. 2018, 2021). This kind of research represents a relatively new area arising from advancements in neuroimaging technologies. These methods have been influential because they allow researchers to examine how different brain regions communicate during rest, providing insights into the brain's functional architecture and its alterations under various conditions (Mirabella et al. 2021). RsfMRI has been widely used in preclinical studies of various diseases, such as depression, autism spectrum disorder (ASD), Huntington's disease (HD), and Parkinson's disease (PD) (Lv et al. 2018; Pagani et al. 2023). Despite its potential, there has been limited evidence for applying rsfMRI in studying the effects of IMF on aging brains.

However, rsfMRI provides only a broad, high‐level perspective on the variations in cerebral function across the entire brain. To further explore the variation of the cerebral function, other studies related to the neural function were necessary. For example, myelination, the process of forming myelin sheaths around axons, is essential for proper neuronal function and is significantly affected by aging (Almeida and Lyons 2017; Nickel and Gu 2018). Proteins such as myelin basic protein (MBP) and myelin‐associated glycoprotein (MAG) are critical in the formation and stability of myelin (Voskuhl et al. 2019; Deng et al. 2023). Recent theoretical developments suggest that dietary interventions, including caloric restriction and nutritional therapy, can influence myelination (Langley, Triplet, and Scarisbrick 2020; Zarini 2021). Increased expression of MBP and MAG has been observed in response to similar therapies, indicating enhanced myelin integrity and repair (Zhang et al. 2019; Clavreul, Dumas, and Loulier 2022). However, there has been limited evidence regarding the specific impact of IMF on these myelin‐related proteins in the context of aging.

Therefore, dietary interventions are common in aging research, where the integration of advanced imaging techniques with behavioral assessments remains limited. This gap hinders a complete understanding of the complex interactions between dietary interventions and brain health. Therefore, our study aims to address these gaps by employing a comprehensive experimental design that includes behavioral tests, neuroimaging, and ultrastructural analysis to assess the effects of IMF on physical and cognitive functions in aged mice. Here, we utilized a comprehensive approach to investigate the impact of IMF on the aging brain, including small animal MRI (rsfMRI), EEG/EMG recordings, transmission electron microscopy (TEM), and immunohistochemistry (IHC). The combined application of these techniques has been influential because they provide a holistic view of how dietary interventions can affect brain health and function (Whittaker et al. 2023; Kapogiannis et al. 2024). By exploring these aspects in detail, this study aims to provide valuable insight into the potential of IMF as a dietary intervention to mitigate age‐related somatic function decline and promote healthy aging.

## 2 Materials and Methods

This study aimed to comprehensively evaluate the behavioral, neural, neuroimaging, and histological changes in aged mice using a combination of behavioral tests, imaging techniques, and histological analyses. The following sections detail the experimental procedures and methodologies used, including animal handling, behavioral assessments, imaging and electrophysiological data acquisition, and histological techniques.

### 2.1 Mice

All procedures were approved by the Animal Care and Use Committee at the Innovation Academy for Precision Measurement Science and Technology, Chinese Academy of Sciences. Wild‐type male C57BL/6 mice (15 months old) were purchased from Hunan SJA Laboratory Animal (Hunan, China). All animals were housed in a temperature‐controlled environment with a constant 12‐h light/dark cycle and were randomly assigned to experimental groups. Water was available *ad libitum*. Food was only freely available in the IMF group from 9 AM to 3 PM for 10 weeks.

### 2.2 Behavioral Tests

To comprehensively evaluate the motor, spatial exploration, and coordination abilities of aged mice, several behavioral tests were conducted, including the open‐field test, Y‐maze test, wire hanging test, and beam‐walking test. These tests were designed to assess various aspects of physical and cognitive functions in the experimental animals.

#### 2.2.1 Open‐Field Test

The open‐field test was used to explore the motor ability of aged mice. The open‐field test was conducted in an open‐field apparatus (40 × 40 × 40 cm). The apparatus was divided into a center zone and an outside zone. The center zone is a square, 10 cm away from the wall. The rest of the apparatus, except for the center zone, is defined as the outside zone. Each animal was placed in the outside zone at the beginning. Each test was recorded for 5 min, and the total distance traveled and average speed were analyzed using Any‐maze software.

#### 2.2.2 Y‐Maze Test

The novel arm exploration experiment was used to detect the spatial exploration ability of aged mice. A Y‐shaped apparatus was used, consisting of three symmetrical arms at a 120° angle (30 cm long, 7 cm wide, and 15 cm high). The three arms of the Y maze are named the start arm, the novel arm, and the other arm, respectively. The experiment was divided into training and testing periods. During the training period, the novel arm was blocked with a partition, and the mice were placed in the start arm and allowed to move freely between the start and other arms for 10 min. After training, the mice were returned to their cage. The test period was conducted 2 h later. During the test period, the partition blocking the novel arm was removed, and the mice were placed in the start arm and allowed to move freely among the three arms for 5 min. The entire experimental process was video recorded and analyzed using Any‐maze software. Any‐maze software started timing once it recognized the mouse placed in the start arm. The software performed quantitative analysis, recording the time spent and the number of entries into each arm.

#### 2.2.3 Wire‐Hanging Test

The wire‐hanging test assesses experimental animals' upper limb strength and motor coordination. The mice were suspended on a wire 24 cm above the ground. Timing begins when the mouse is successfully suspended for the first time. After the mouse falls, it is quickly suspended again. The entire experiment was recorded. The total time the mouse hung on the wire and the number of falls within 3 min were counted.

#### 2.2.4 Balance Beam Test

The beam‐walking test is used to evaluate the fine coordination and balance abilities of mice. The balance beam is 1 m long, 6 mm or 12 mm wide, and 50 cm high from the ground. There is a light placed at the starting point of the balance beam as an aversive stimulus for the mice; a black box is placed at the endpoint to attract the mice during the training stage, but it is removed during the testing phase to prevent interference. The experiment is divided into two stages: the training stage and the testing stage. During the training stage, mice need to successfully pass the center area of the 6 mm balance beam (80 cm) twice to be considered successfully trained and eligible to enter the testing stage. During the testing stage, the time it took for the mice to pass through the center area of the 6 mm balance beam once was recorded. The testing process was recorded by a camera. The test was repeated twice, and the average of the passing times was taken as the result.

### 2.3 Magnetic Resonance Imaging and Electrophysiology

To further investigate the neural correlates underlying behavioral changes, rsfMRI and EEG/EMG data were acquired from the mice. These imaging and electrophysiological techniques provided comprehensive insight into the brain's functional connectivity and muscle activity.

#### 2.3.1 Data Acquisition of rsfMRI

In this study, functional magnetic resonance imaging (fMRI) experiments were conducted on a Bruker 7T MRI scanner (Bruker Bio Spin, Germany) using a mouse head cooling coil (MRI Cryo Probe, Bruker, Germany). The detailed procedures can be found in our former studies, here only a brief procedure was provided. There were nine animals involved in every group, and one mouse was excluded from each group due to fixation issues, which affected the image quality. The animal was initially anesthetized with 3.0% to 4.0% isoflurane, followed by an intraperitoneal injection of dexmedetomidine hydrochloride (0.1 mg/kg/h). Next, the mice were secured to the MRI animal bed via ear and bite bars. The cradle was then transferred to the magnet. The isoflurane concentration was adjusted based on the animals' physiological responses to maintain light anesthesia and 0.1 mg/kg/h dexmedetomidine hydrochloride was continuously infused after the bolus injection via a subcutaneously implanted needle for no longer than 1 h to minimize mouse movement during scanning to maintaining the physiological stability. A circulating water bath system maintained the mice's rectal temperature at 37°C ± 0.5°C. The physiological parameters were monitored using a small animal monitoring system (Model 1025; Small Animal Instruments Inc.) and a Dräger Infinity Delta monitor, which included rectal temperature probes, heart rate sensors, and respiratory rate sensors. The inhalation concentration of isoflurane was adjusted to keep the respiratory rate between 75 and 100 breaths per minute.

During the acquisition procedure, global shim optimization of magnetic field homogeneity was initially performed, followed by the local second‐order shim optimization using the MAPSHIM protocol. The anatomical images were acquired using a Turbo‐RARE sequence with the following parameters: field of view (FOV)—12 × 10 mm^2^, matrix size—180 × 180, RARE factor—4, repetition time (TR)/echo time (TE)—5000/8.6 ms, averages—8, flip angle—180°, and slice thickness—0.5 mm. In the Core Scanning area, local field homogeneity was optimized using a pre‐scanned field map. Functional images were acquired using single‐shot echo planar imaging (EPI) with the following parameters: TR—1000 ms, TE—18 ms, segments—2, flip angle—70°, matrix size—70 × 70, nominal in‐plane resolution—200 × 200 μm^2^, slice thickness—500 μm, number of slices—16, and 300 EPI volumes (10 min). All 300 volumes were acquired in a single EPI scan.

### 2.4 EEG/EMG Data Acquisition and Analysis

Mice were anesthetized with 1% sodium pentobarbital (100 mg/kg, intraperitoneally) and subsequently secured on a mouse brain stereotaxic frame. A skin incision exposed the skull, enabling the implantation of four metal skull screws bilaterally in the frontal cortex and other cortical regions for electroencephalographic (EEG) recordings. Additionally, two Teflon‐coated electromyography (EMG) electrodes were implanted into the neck muscles bilaterally to capture muscular activity. These electrodes were connected to a six‐pin plastic plug, soldered in place, and affixed to the skull using dental acrylic resin. The animals were allowed a recovery period of 7 days prior to further experiments. To investigate the impact of IMF on sleep patterns, continuous 24‐h recordings of EEG and EMG signals from mice were conducted using in vivo electrophysiological techniques. The EEG and EMG signals were concurrently acquired using the Medusa Small Animal Electrophysiology Recording System (Medusa, Bio‐Signal Technologies, Nanjing, Jiangsu, China), sampled at a frequency of 1000 Hz and filtered at 50 Hz to eliminate noise.

Custom‐written software was employed for the analysis of both EEG and EMG data. To ensure the accuracy of data extraction, synchronized video recordings were used to identify wakeful periods, during which EEG validation confirmed a task‐free, freely moving state. EMG data from these periods were analyzed using signal processing techniques such as root mean square (RMS) calculations, continuous wavelet transform (CWT), and power spectral density (PSD) analysis. These methods allowed for the quantification of signal amplitude, energy, frequency content, and power distribution.

### 2.5 Histological Analysis

To complement the behavioral and imaging studies, histological analyses were performed, including transmission electron microscopy (TEM), hematoxylin & eosin staining, and immunohistochemistry. These analyses provided detailed insights into the cellular and molecular changes in the brain and peripheral tissues.

### 2.6 Transmission Electron Microscopy (TEM) Methodology and Analysis

For transmission electron microscopy (TEM), animals were perfused transcardially and sacrificed. Brain tissues were fixed in PBS containing 1.25% glutaraldehyde and 2% paraformaldehyde, followed by post‐fixation with 1% OsO_4_ in phosphate buffer (PB) at room temperature (20°C) for 2 h. Tissue blocks, less than 1 mm^3^ in size, were then dehydrated through a graded ethanol series (50%, 70%, 80%, 90%, 95%, and 100%, each for 15 min) and embedded in resin blocks. These blocks were subsequently cut into 60–80 nm ultrathin sections and stained with 2% uranyl acetate and lead citrate, each for 15 min. Sections were air‐dried at room temperature overnight. Images were captured using a transmission electron microscope (Hitachi, HT7700). The inner and outer diameters of the myelin sheath were measured using ImageJ. The g‐ratios, representing the ratio of the inner axonal diameter to the total outer diameter of the myelin sheath, were used as a robust measure of myelination degree. The g‐ratios of myelinated fibers in the corpus callosum (CC) and medulla oblongata (MO) were calculated as the ratio of the inner diameter to the outer diameter of the myelin sheath.

### 2.7 Hematoxylin & Eosin Staining

Mice were anesthetized using 1% sodium pentobarbital, followed by perfusion with electron microscopy fixative through the heart. The forelimb muscles were dissected out. The excised muscle samples were embedded and cryosectioned to produce sections of 4 μm thickness. Hematoxylin solution was applied for 3–8 min to stain the cell nuclei, followed by rinsing with tap water. This was followed by a 1–3 min incubation with eosin solution, which was also rinsed with tap water. Dehydration and clearing were achieved using graded ethanol and xylene. Sections were mounted with neutral resin. Images of H&E‐stained sections were captured using a microscope (Nikon Eclipse CI).

### 2.8 Immunohistochemistry

Mice were similarly anesthetized with 1% sodium pentobarbital and underwent cardiac perfusion with PBS followed by 4% paraformaldehyde. After perfusion, brain tissue was collected. The brain tissue underwent graded dehydration before being sectioned at a thickness of 40 μm using a cryostat. Tissue sections were subjected to immunostaining with primary antibodies against MBP (rabbit, 1:200), MAG (rabbit, Proteintech, Catalog Number: 14386‐1‐AP, 1:500), PLP1 (rabbit, Abclonal, Catalog Number: A15100, 1:200), and SOX10 (rabbit, Abclonal, Catalog Number: A15100, 1:200). Incubation occurred overnight at 4°C. The secondary antibody used was Goat Anti‐Rabbit Cy3 (1:400), incubated at room temperature for 1 h. Nuclei were counterstained with DAPI (1:5000). After thorough washing with PBS and air‐drying, sections were mounted with coverslips using 70% glycerol. All wash steps were conducted using PBS. Slides were stored at 4°C. Fluorescent imaging and confocal microscopy were performed using an Olympus VS200 and SP8 Leica confocal microscope.

### 2.9 fMRI Data Preprocessing

All fMRI data were processed using the AFNI (Analysis of Functional NeuroImages), FSL (Analysis Group, FMRIB, Oxford, UK), and ANTS (Advanced Normalization Tools) software packages (available on GitHub at https://github.com/ANTsX/ANTs), along with MATLAB scripts. MRI data were initially converted to NIFTI format using Bru2Nii (Bruker, Germany), followed by semi‐manual skull stripping of T2 images using ITK‐SNAP (http://www.itksnap.org/). Brain extraction and masking were then performed using fslmaths in the FSL software, followed by registration of EPI to T2 images using antsRegistrationSyN.sh in the ANTS software package (http://picsl.upenn.edu/software/ants/), with subsequent application of N4BiasFieldCorrection and Denoise Image for image quality enhancement. Subsequently, brain regions were segmented using a study‐specific mouse template (https://atlas.brain‐map.org/) for group analysis, and masks were adjusted to match the EPI images to ensure anatomical consistency. Time‐series data were then corrected for motion and temporal shifts using ants Motion Corr and 3dTshift in AFNI. Finally, all animals' EPI images were spatially normalized to the TMBTA mouse brain template using linear and nonlinear transformations to facilitate anatomical localization, and the normalized images were spatially smoothed using a Gaussian kernel with a full width at twice the voxel size (0.4 mm isotropic Gaussian kernel). Time‐series extraction for the ROIs was then performed on the images registered to the TMBTA standard template using AFNI's 3dNetCorr tool, facilitating subsequent analyses similar to our previous studies on functional networks.

### 2.10 Functional Connectivity Analysis

A total of 31 regions of interest (ROIs) were manually merged based on the Allen Mouse Brain Common Coordinate Framework v3 (CCFv3) template using FSL (Wang et al. 2020). This included regions in the isocortex, olfactory areas, hippocampal formation, cortical subplate, striatum, pallidum, thalamus, hypothalamus, midbrain, and pons (Figure S1A). Table S1 lists all ROIs. An ROI‐averaged time series was generated for each ROI by averaging the fMRI data (smoothed residuals) across all voxels encapsulated by the ROI using the 3dNetCorr tool. Functional connectivity matrices were constructed by calculating the Pearson correlation coefficient (using Python) between all responsive ROIs (*n* = 31) ipsilateral to the stimulation site, resulting in a 31 × 31 connectivity matrix for each animal. These matrices were Fisher‐z transformed for group averaging and statistical tests, with results converted back to *r* for presentation. The average matrix for each group was computed by averaging the individual matrices. To identify significant connectivity differences between the two groups, a two‐sample *t*‐test with family‐wise error (FWE) correction was performed on the matrices, resulting in brain region pairs with significant differences (*p* < 0.05). The significant brain region pairs were visualized using a chord diagram to illustrate the whole‐brain network changes. Additionally, scatter plots for each significantly changed brain region pair were generated, displaying the distribution of connectivity values and the results of the two‐sample t‐tests.

### 2.11 Statistical Analysis

Statistical details for each experiment are provided in the figure legends, with “*n*” representing the number of animals per group. Statistical significance was defined as *p* < 0.05.

## 3 Result

**FIGURE 1:**
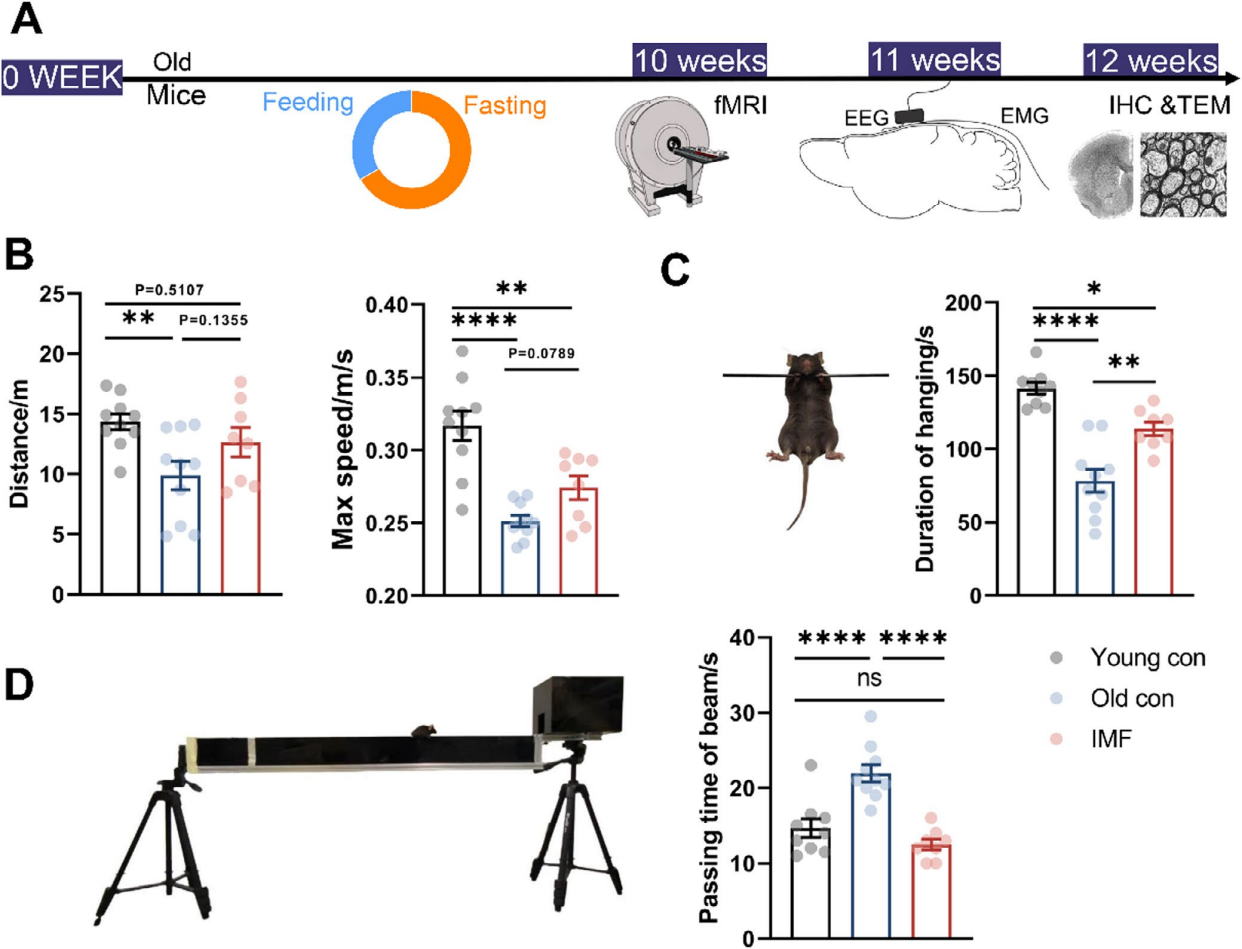
Intermittent fasting (IMF) positively impacts various aspects of physical in aged mice. (A) Flowchart of the experimental design, and the pie chart depicts the schedule of feeding (blue) and fasting (orange) phases; Behavioral test statistics and schematic diagram; (B) Distance traveled and Max speed for the open‐field test; (C) Duration of sustained hanging in 3 min for the hanging wire test; (D) Completion time of balance beam test. Young control, *n* = 10; Old control, *n* = 10; IMF, *n* = 8; Data were represented with mean ± SEM; *p < 0.05, **p < 0.01, ****p < 0.0001, two‐way ANOVA.

In this study, we implemented a 10‐week intermittent fasting (IMF) regimen for the aging animals in the experimental groups, roughly equivalent to 2 years of human health management (Figure 1A). The timeline outlines the experimental design, showing that the old mice underwent a feeding and fasting schedule over this period. Key assessments, including small animal MRI (rsfMRI), EEG/EMG recordings, transmission electron microscopy (TEM), and immunohistochemistry (IHC) analyses, were conducted at the end of the fasting weeks.

### 3.1 IMF Positively Impacts Various Aspects of Motor Coordination in Aged Mice

As a nutritional intervention, IMF has shown promising results in enhancing emotional learning and memory (Tang et al. 2023; Whittaker et al. 2023). To explore its impact on aged animals, we conducted several behavioral assessments on aged mice. The open‐field test (Figure 1B) was used to measure the distance traveled by the mice, indicating their locomotor activity and exploratory behavior. There is a significant decrease in the distance and Max speed for the old control group comparing to the young control group (*p* < 0.01). Although the IMF group displayed reduced locomotor activity compared to the young control group (*p* < 0.01), there were potential improvements in motor function compared to the old control group (*p* < 0.05). Representative images of the routes taken by the mice are shown in Figure S1.

Furthermore, former studies also highlighted that IMF can enhance motor coordination, balance, and muscle strength, contributing to better overall physical health (Longo Valter and Mattson Mark 2014; Longo and Panda 2016; Brandhorst et al. 2024). Therefore, IMF has been linked to various physical improvements, particularly in aged populations. To further explore the specific physical improvements brought about by IMF, the hanging wire test (Figure 1C) measured muscle strength and endurance. The IMF group also showed a significantly longer duration of hanging compared to the old control group, confirming improved muscular strength and endurance (*p* < 0.01). Additionally, the balance beam test (Figure 1D) was used to evaluate motor coordination and balance. Mice in the IMF group demonstrated a significantly shorter time to traverse the balance beam compared to the old control group, indicating superior motor coordination and endurance (*p* < 0.001). They also showed comparable motor abilities to the young control group. These findings aligned well with the previous studies suggesting that IMF can improve physical performance and muscle function in older adults (Egimendia et al. 2019; Bang et al. 2022). However, we did not find any significant difference for these three groups in the Y‐maze test (Figure S2).

Our results indicated that IMF could positively impact various aspects of motor coordination, balance, and muscle strength functions in aged mice. Therefore, IMF could enhance physical performance by improving motor coordination and muscle strength through specific physiological mechanisms.

### 3.2 Effects of IMF on Muscle Activity

**FIGURE 2:**
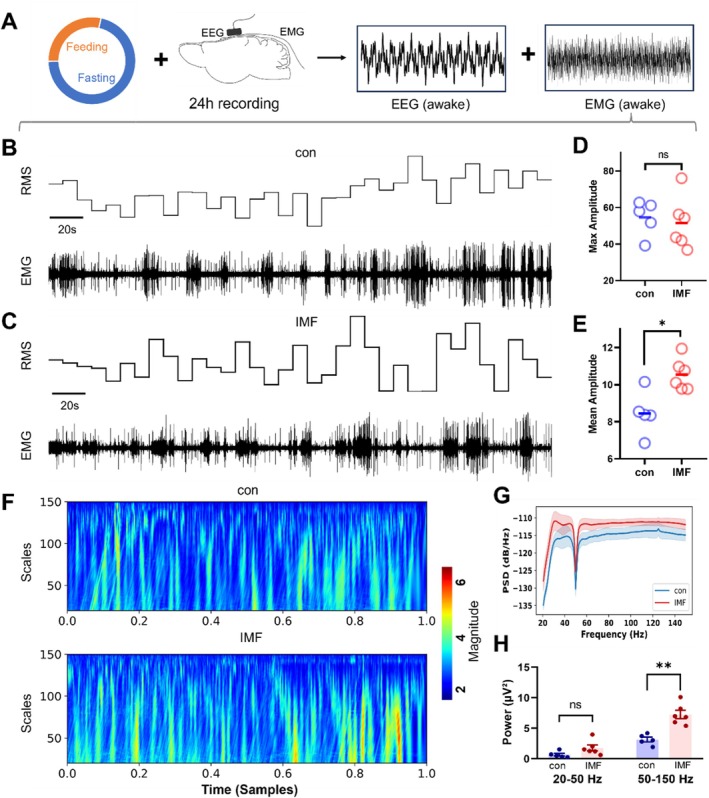
Effects of intermittent fasting (IMG) on EMG activities and sleep patterns. (A) Schematic representation of the experimental design; EMG activities during the feeding period. Raw EMG signals and the root mean square (RMS) of the EMG signals are presented for the control (B) and IMF (C) groups during the wakeful period (representative traces, 300 s, amplitude bar marked on the side); The maximum amplitude (D) and the average amplitude (E) of EMG signals during the wakeful period; (F) Representative time‐frequency plots for EMG for each group; (G, H) Power spectral density (PSD) analysis of EMG signals and their corresponding segmented statistical results. Data represent mean ± SEM. *p < 0.05, **p < 0.01, two‐sample *t*‐test.

To comprehensively evaluate the motor abilities of IMF animals, we conducted a 24‐h EMG recording for the IMF group (*n* = 6) and the control group (*n* = 5). Simultaneously, a 24‐h EEG recording was performed to filter EMG levels during different periods, including wakefulness and sleep. The effects of IMF on muscle activity and performance were analyzed using electromyography (EMG) and power spectral density (PSD) measurements. Figure 2A presents a schematic representation of the experimental design, illustrating the feeding and fasting phases, along with the setup for EEG and EMG recordings over a 24‐h period. It also outlines the steps for extracting and analyzing EMG data during the wakefulness phases. The raw EMG signals and their root mean square (RMS) were analyzed and compared between the IMF and control groups (Figure 2B–E). The RMS values (Figure 2D,E) indicated that, while the maximum amplitude of the EMG signals showed no significant changes, the average amplitude in the IMF group was significantly higher than in the control group (*p* < 0.01), suggesting that IMF could enhance muscle activity.

Additionally, power spectral density (PSD) analysis was conducted to further assess muscle activity. Results showed that the IMF group had higher PSD values in the basic motor‐related frequency range (20–150 Hz) compared to the control group (Figure 2G,H) with a significant enhancement in the higher frequency range (50–150 Hz). Representative time‐frequency plots are shown in Figure 2F. These findings indicated that basic muscle activity, reaction speed, and contraction force were enhanced in the IMF group (Osanai, Yamamoto, and Kitamura 2023).

These results support our behavioral findings, suggesting that IMF improves physical performance and muscle function. We hypothesize that IMF enhances the physiological states of the muscles, thereby improving motor abilities. While improvements in brain function during IMF have been widely studied, the specific brain regions responsible for these changes in muscle function remain unidentified (Anton et al. 2018; Orfanos et al. 2018). To pinpoint the neural mechanisms behind these improvements, we have designed new experiments aimed at identifying the brain regions involved in regulating these observed benefits.

### 3.3 Functional Connectivity Analysis

Resting‐state functional magnetic resonance imaging (rsfMRI) is widely used to explore brain connectivity and its alterations under various conditions, including aging and dietary interventions such as IMF. Additionally, it has the potential for cross‐species applications, making it suitable for broad research on fasting (Chechko et al. 2015; Pagani et al. 2023). This technique's ability to capture whole‐brain network interactions provides valuable insights into the neural mechanisms underlying behavioral changes. Studies using rsfMRI have demonstrated its potential to reveal network‐level abnormalities associated with different diseases, highlighting the diverse and robust nature of this imaging modality (Pagani et al. 2021; Zerbi et al. 2021).

**FIGURE 3:**
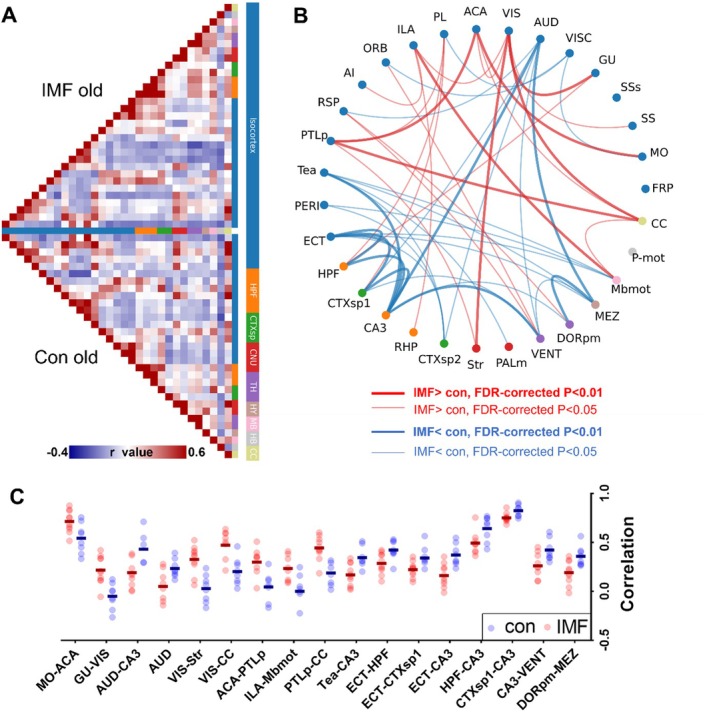
Functional connectivity (FC) analysis of animals in different groups. (A) FC matrices for aged control (Con) and intermittent fasting (IMF) groups (red for positive correlation, blue for negative correlation); (B) Network graph displaying significant differences in FC between aged control and IMF groups. Red edges indicate stronger connections in the IF group, blue edges indicate stronger connections in the control group; (C) Distribution of significant different correlation values (*p* < 0.01) for FC between different brain regions in control (CON) and IMF groups. Abbreviations and corresponding full terms are listed in the Table S1. Two‐Sample *t*‐Test, FDR‐corrected.

To explore the impact of IMF on brain‐wide functional connectivity (rsFC), we compared the rsFC between aged control (Control group) and IMF mice using rsfMRI. Based on the Allen Brain Atlas, the whole brain was divided into nine major regions and further subdivided into 31 subregions, defined as regions of interest (ROIs) to calculate pairwise correlation matrices. Intergroup comparison of the whole‐brain pairwise correlation matrices revealed significant changes in the brain‐wide rsFC of IMF mice compared to Con old mice (Figure 3A,B). This analysis identified 17 brain region pairs with significant differences (*p* < 0.01, FDR‐corrected), which are highlighted in Figure 3B. These region pairs (Figure 3C) include seven with significantly enhanced connectivity (MO‐ACA, GU‐VIS, VIS‐Str, ACA‐PTLp, VIS‐CC, PTLp‐CC, and ILA‐Mbmot) and ten with significantly reduced connectivity (AUD‐CA3, AUD‐Tea‐CA3, ECT‐HPF, ECT‐CTXsp1, ECT‐CA3, HPF‐CA3, CTXsp1‐CA3, CA3‐VENT, and DORpm‐MEZ). These findings suggest that IMF leads to a reorganization of brain connectivity, which might underlie the observed improvements in motor functions (Papalini et al. 2024).

The specific brain regions affected by IMF, particularly those related to motor and sensory processing, highlight the potential mechanisms through which IMF exerts its benefits. These results underscore the need for further experiments to delineate the exact brain regions and pathways involved in these improvements. Identifying the specific brain areas influenced by IMF will provide a deeper understanding of the neural basis of IMF's effects on aging and pave the way for targeted interventions to promote healthy aging (De Cabo and Mattson 2019; Brandhorst et al. 2024). Specific *p*‐values are provided in Table S2. In summary, the rsfMRI data supports the behavioral findings, indicating that IMF enhances brain connectivity in specific regions, which may contribute to the improved motor function observed.

### 3.4 Whole‐Brain Functional Connectivity Analysis

**FIGURE 4:**
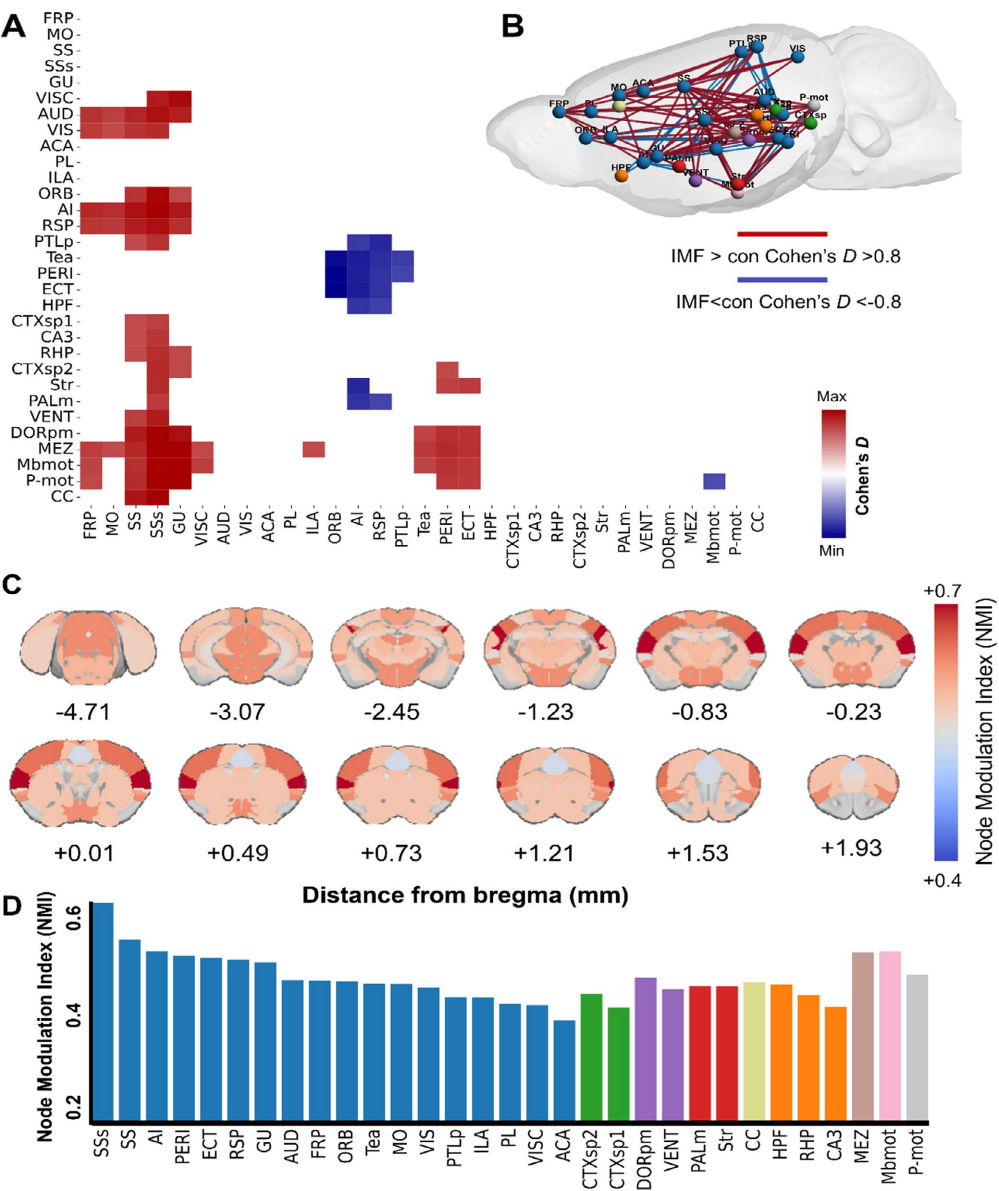
Whole‐brain Cohen's *D* effect size analysis in aged control and intermittent fasting (IMF) groups. (A, B) Effect size matrices and network graph displaying significant differences between aged control and IMF groups; (C) Node Modulation Index (NMI) visualization: These highlights regions with significant changes in connectivity strength between the aged control and IMF groups; (D) NMI distribution for specific brain regions: This distribution underscores which brain areas exhibit the most pronounced connectivity changes under intermittent fasting conditions.

To address the limitations inherent in traditional functional connectivity analyses, we extended our study using Cohen's *D* effect size (Lombardo et al. 2016; Bowring et al. 2021; Han et al. 2022). This approach enhances the precision of our findings by quantifying the magnitude of connectivity changes rather than merely identifying their presence. Figure 4A illustrates the matrix of brain region pairs with effect sizes greater than 0.8, indicating large effect sizes. The visualization of these pairs in a 3D ball‐and‐stick model (Brainnet Viewer) highlights significant connectivity changes, with red indicating stronger connectivity in the IMF group (Cohen's *D* > 0.8) and blue indicating stronger connectivity in the control group (Cohen's *D* < −0.8).

Eighteen ROIs exhibited strong effects with a Cohen's *D* greater than 0.8, indicating significant modulation in connectivity. The most pronounced variations were observed in these brain regions. These areas were ranked based on the overall connectivity changes linked to each ROI, quantified by the NMI from the mean effect size (Cohen's *D*) of resting‐state functional connectivity change. The NMI serves as a surrogate marker, derived by averaging the effect size of connectivity changes across the top 10% of connections for each brain area of interest (Lombardo et al. 2016; Sasai et al. 2021).

The distribution of NMI values for different brain regions, as shown in Figure 4B,C, highlights that the strongest changes occurred in the somatosensory cortex (SSs), mediodorsal thalamus (MEZ), motor‐related midbrain (Mbmot), and other regions closely associated with motor and sensory functions. Figure 4C reveals the NMI values mapped onto brain slices at various distances from the bregma, providing a comprehensive visualization of how IMF differentially impacts specific brain regions. This detailed mapping offers a nuanced understanding of IMF's influence on brain connectivity (Zerbi et al. 2021).

Figure 4D presents bar plots representing all ROIs. These findings support the hypothesis that IMF induces distinct modifications in brain connectivity patterns, as demonstrated in Figure 3B,C, which may underlie the observed improvements in physical performance. To further validate these results, additional experiments are needed to pinpoint the exact brain regions and mechanisms responsible for these changes, as indicated by the significant connectivity alterations identified in our study (Figure 4C,D).

### 3.5 Seed‐Based Functional Connectivity Analysis

**FIGURE 5:**
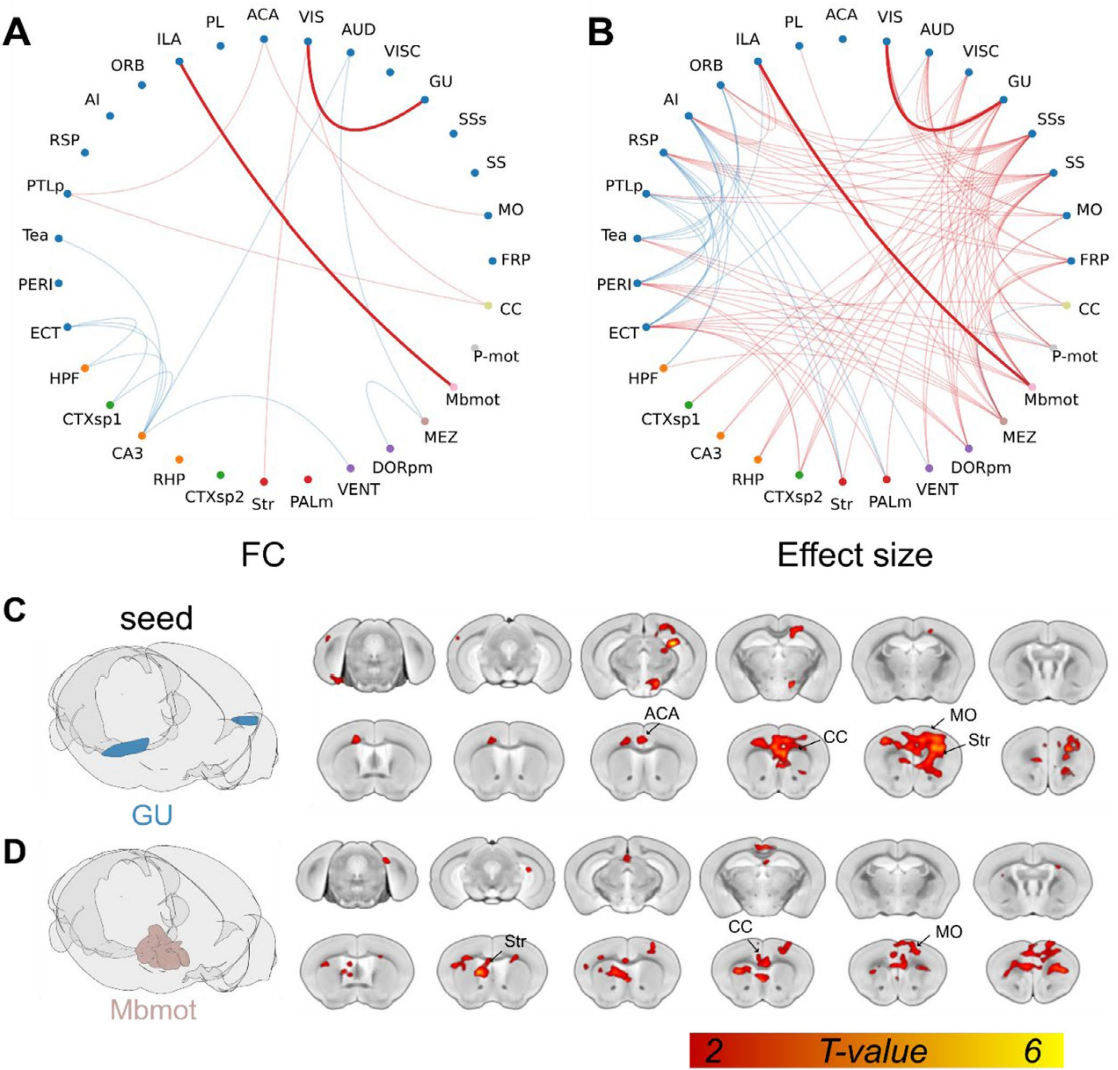
Voxel‐by‐voxel functional connectivity analysis of seed. (A, B) Chord plots of highly significant changes in functional connectivity and corresponding effect size changes; (C, D) Seed‐based connectivity maps for the regions of gustatory cortex (GU) and motor‐related midbrain (Mbmot). The left panel shows the 3D plot indicating the seed location. The right heatmap areas denote regions with significant connectivity differences (*t*‐value) compared to the control group.

Based on the findings from the whole‐brain functional connectivity analysis, we performed multiple screenings of all brain regions based on FC and effect size (Figure 5A,B). The brain regions with significant changes (*p* < 0.01) shown in Figure 1C are all depicted in Figure 5A, with the corresponding changes in the effect size of the connections displayed in Figure 5B. We identified the GU‐VIS and Mbmot‐ILA connections as having significant and substantial differences. Following these results, we conducted seed‐based connectivity analysis maps for GU and Mbmot, which were analyzed and displayed in Figure 5C,D. The left panel shows the 3D plot indicating the seed locations. On the right, the heatmap areas denote regions with significant connectivity differences (*t*‐value) compared to the control group, with the aforementioned four seed points. The connectivity changes are visualized across multiple coronal slices. We found that the significant network changes identified in these analyses pointed to several common regions, primarily concentrated in the motor cortex (MO) (McColgan et al. 2020) and corpus callosum (CC) (Smith, Mayer, and Duncan 2013; Petrus et al. 2019), which are well‐studied areas related to motor abilities. We found that the functional connectivity strength of the pathways identified through multi‐parameter screening exhibited varying degrees of strong positive correlations with the hanging time shown in Figure 1C, and moderate to strong negative correlations with the passing time parameter shown in Figure 1D. These results suggest that improvements in motor performance are well reflected in the motor‐related brain networks within the resting‐state functional connectivity (Figures S3 and S4).

### 3.6 Transmission Electron Microscopy (TEM) Methodology and Analysis

**FIGURE 6:**
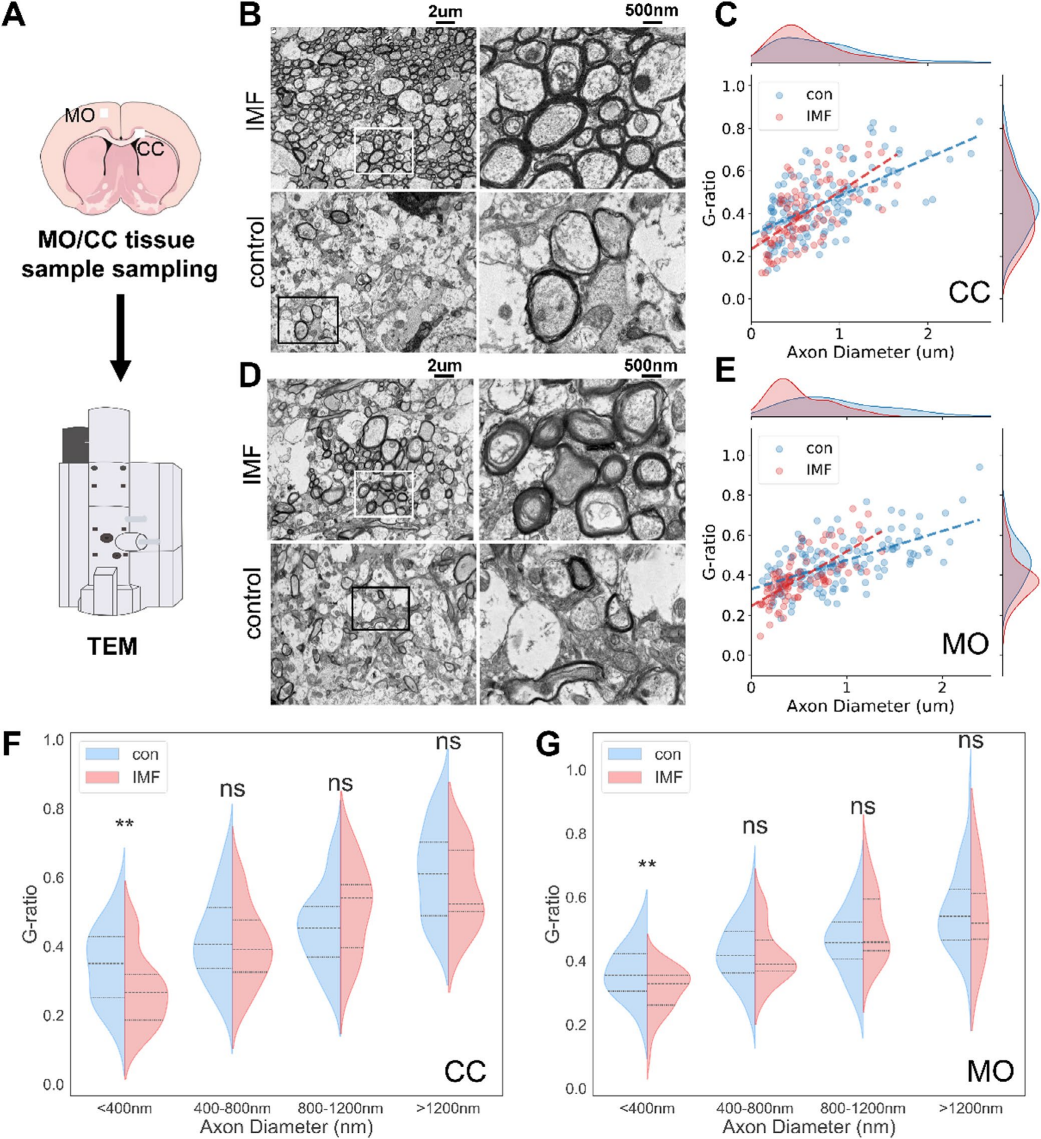
Transmission Electron Microscopy (TEM) methodology and analysis. (A) Outline of the tissue collection strategy following completion of fMRI scans; (B) TEM images showing the ultrastructure of axons in the corpus callosum (CC). The right side shows the corresponding detailed views of axon myelination; (C) G‐ratio versus axon diameter scatter plot for CC region. Each point represents an individual axon. The density distribution along the margins shows the distribution of G‐ratios and axon diameters for each group; (D) TEM images showing the ultrastructure of axons in the motor cortex (MO). The right side shows the corresponding detailed views of axon myelination; (E) G‐ratio versus axon diameter scatter plot for MO region. The density distribution along the margins shows the distribution of G‐ratios and axon diameters for each group. (F, G) Comparative analysis of G‐ratios on axons with different diameters in the CC and MO regions between the control (con) and intermittent fasting (IMF) groups. The trend lines indicate the linear relationship between these two metrics. Violin plots represent mean ± SEM; **p < 0.01, two‐sample *t*‐test.

To investigate the specific brain regions influenced by IMF identified in our previous screening, we focused on the corpus callosum (CC) and motor cortex (MO) (Figure 6A). Using TEM, we analyzed the ultrastructure of axons in these regions (Figure 6B,D). The inner and outer axonal areas were segmented to calculate their circular equivalent diameters, determining axon and myelin thickness. Additionally, the G‐ratio, a robust measure for assessing the degree of axonal myelination, was calculated (Chomiak and Hu 2009; Kaiser et al. 2021).

In the CC, density distribution maps showed a significant reduction in axon diameters in IMF mice compared to control mice, indicating higher axonal degeneration in the IMF group (Figure 6B). The G‐ratio analysis plot (Figure 6C,F) revealed differences in myelination between the groups, with IMF mice exhibiting a decreased G‐ratio in axons with diameters < 400 nm, indicating thicker myelin. This increase in myelin thickness was particularly pronounced in smaller‐diameter axons.

Similarly, in the MO region, TEM images revealed differences in axon and myelin morphology between IMF and control mice (Figure 6D). The G‐ratio analysis plot (Figure 6E,G) showed a significant decrease in the G‐ratio in IMF mice compared to control mice within the range of axons smaller than 400 nm, indicating increased myelin thickness. Consistent with the findings in the CC region, this effect was more pronounced in smaller diameter axons.

These changes often occur during the treatment or recovery from demyelinating diseases, including MS recovery, aging improvement, and HD recovery. These findings suggest that IMF induces changes in axonal myelination in both the CC and MO regions, which may underlie the observed alterations in functional connectivity. Evidence for these relationships is sparse in rodent studies, with most research focusing on humans and primates (Lv et al. 2018; Langley, Triplet, and Scarisbrick 2020).

The changes in myelination could contribute to the enhancements in motor function performance previously observed in IMF‐treated mice. There have been some reports on the improvement of myelination with IMF (Langley, Triplet, and Scarisbrick 2020; Zarini 2021). Given the critical role of myelin in neuronal function and signal transmission, these results underscore the potential of IMF as a strategy for improving neuronal health and function.

To further elucidate the mechanisms underlying these changes, our subsequent analyses will focus on the levels of specific myelin‐related proteins, such as myelin basic protein (MBP) and myelin‐associated glycoprotein (MAG), in the CC and MO. This will provide a more detailed understanding of how IMF impacts cellular and molecular processes associated with myelination and motor function.

### 3.7 Immunohistochemical Analysis of MBP and MAG Expression in Brain

**FIGURE 7:**
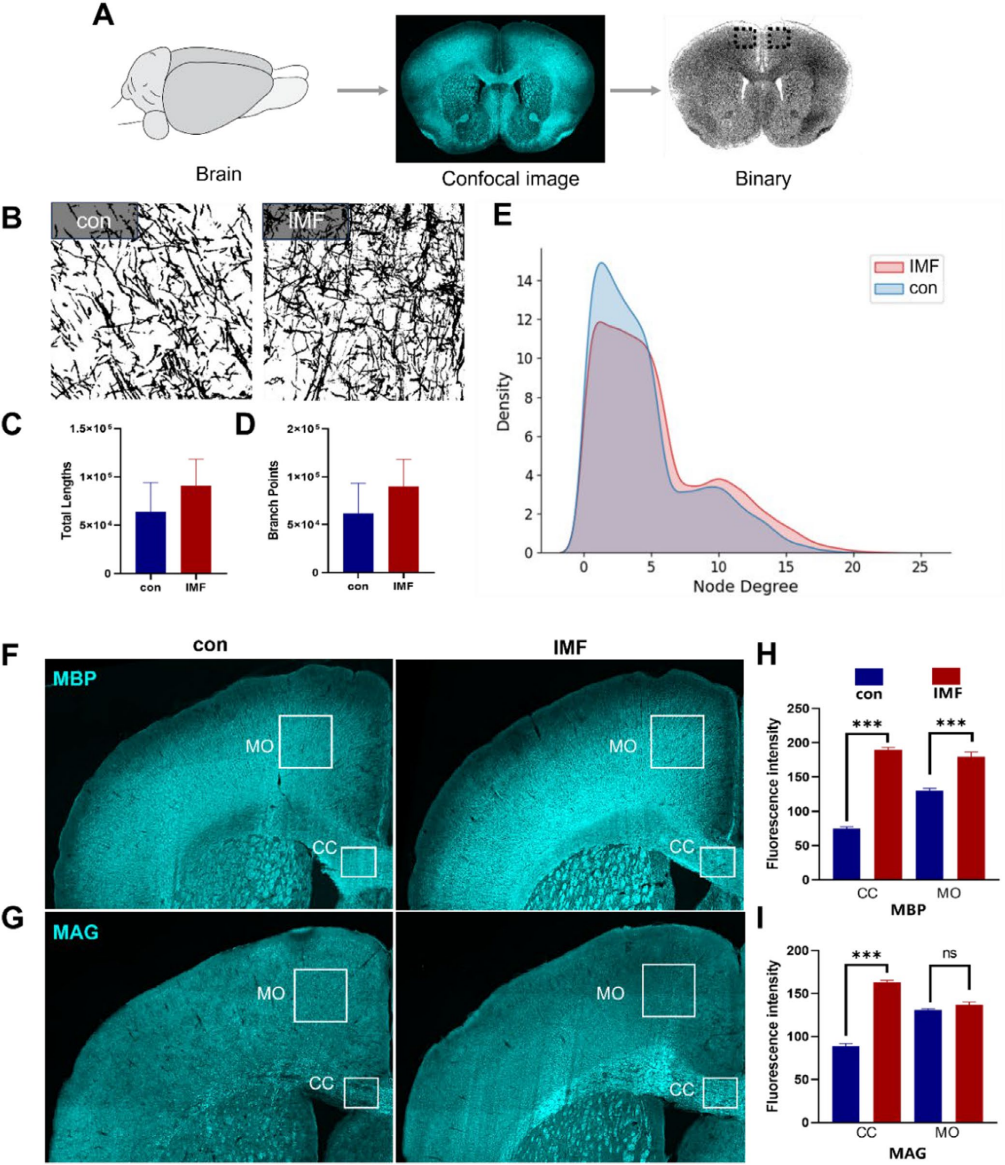
Immunohistochemical analysis of myelin basic protein (MBP) and myelin‐associated glycoprotein (MAG) expression. (A) Workflow for confocal image acquisition and processing. Outlines the steps involved in capturing and analyzing confocal images to assess myelin integrity; (B–D) Gray‐scale images depicting total fiber length and the number of branch points of myelinated fibers in the motor cortex (MO). These metrics provide insight into the structural changes in myelinated fibers associated with IMF; (E) Distribution of node degree density of axonal fibers, representing the connectivity within the axon network; (F–I) Immunofluorescent staining of MBP and MAG in brain slices from control (con) and intermittent fasting (IMF) groups. Bar plots represent mean ± SEM; ***p < 0.001, two‐sample *t*‐test.

The immunofluorescent images of MBP and MAG in brain slices from both control (con) and IMF groups are shown in Figure 7F,G. Quantitative analysis indicates that MBP staining intensity was significantly higher in the IMF group compared to the control group (Figure 7H), signifying enhanced myelination (Han et al. 2022). This finding suggests that IMF promotes myelin formation and stability in the brain, supporting the hypothesis that dietary interventions can positively influence myelin integrity.

The staining intensity of myelin‐associated glycoprotein (MAG) was significantly greater in the IMF group compared to the control group (Figure 7I). However, no significant change in protein expression was observed in the MO. This enhanced expression of MAG suggests improved myelin stability and function due to IMF, similar to previous findings in multiple sclerosis (MS) research (Chapman and Hill 2020). The results for both MBP and MAG collectively support the notion that IMF not only boosts myelin production but also enhances the quality of the myelin sheath. This improvement provides a stronger foundation for neuronal communication and overall brain health.

Figure 7A outlines the workflow for image acquisition and processing. Brain tissues were sectioned, stained, and imaged using confocal microscopy. These images were then converted to binary format for detailed quantitative analysis of myelination patterns and the structural organization of white matter. Figure 7B–D presents the analysis of total fiber length and the number of branch points of myelinated fibers in the MO. Despite the increase in MBP and MAG expression, no significant differences were found in total fiber length or the number of branch points between the control and IMF groups. Figure 7E shows the distribution of node degree density of axonal fibers, representing connectivity within the brain network. This suggests that IMF enhances myelination at a molecular level while significantly altering the overall structural organization of myelinated axons. The IMF group exhibited a different node degree distribution compared to the control group, especially at high node indices, indicating changes in overall brain network connectivity under IMF conditions (Arancibia‐Cárcamo et al. 2017; Berger et al. 2024).

These differences in node degree density suggest potential changes in network efficiency and integration that merit further investigation. This underscores the importance of understanding how IMF can modify brain structure and function at both molecular and network levels.

## 4 Discussion

Our study aimed to investigate the effects of IMF on physical functions in aged mice, focusing on brain connectivity and myelination. We found that a 10‐week IMF regimen significantly improved physically in aged mice. Behavioral tests showed that the IMF group exhibited enhanced improved motor coordination, and increased muscular strength compared to the control group. These improvements suggest that the IMF may help mitigate age‐related declines in these domains. Advanced imaging techniques revealed significant changes in brain structure and function associated with IMF. Specifically, rsfMRI analyses demonstrated that IMF modulates brain‐wide functional connectivity, enhancing communication between key brain regions. We also observed increased expression of myelin‐related proteins such as MBP and MAG, indicating improved myelin integrity and repair in the IMF group. These findings highlight the potential effects of IMF as a dietary intervention to promote brain health and cognitive functions in aging populations.

### 4.1 IMF and Physical Conditions

Our study provides strong evidence that IMF can enhance physical functions in aged mice. The significant improvements observed in the open‐field test, balance beam test, and hanging wire test suggest that IMF promotes better locomotor activity, motor coordination, and muscle strength. These findings are consistent with previous studies demonstrating the beneficial effects of IMF on physical performance in both animal models and human studies (Boujraf et al. 2006; Lundell et al. 2020; Whittaker et al. 2023). For instance, IMF has been shown to improve motor cortex activity and increase blood oxygenation level‐dependent (BOLD) signals during motor tasks in older adults (Lin et al. 2023), aligning with our observations of enhanced motor coordination and strength in aged mice. Actually, we did not observe a clear relationship between IMF and cognition with the Y‐maze test (Figure S2), suggesting that potential interference between behavioral paradigms may require more detailed future research.

### 4.2 IMF and Cerebral Connectivity

The rsfMRI analyses in our study revealed significant alterations in brain‐wide functional connectivity associated with IMF. Specifically, the IMF group exhibited enhanced connectivity between key brain regions, including the primary sensory and somatomotor areas and the prefrontal cortex. Our findings further demonstrate that these changes in functional connectivity underlie the observed improvements in various motor functions (Figures S3 and S4), highlighting the critical role of efficient interregional communication in supporting optimal brain performance. The findings of altered functional connectivity align with previous research demonstrating the impact of dietary interventions on brain network organization and motor function (Boujraf et al. 2006; Lin et al. 2023).

The rsfMRI study process emphasizes the importance of using resting‐state multi‐parameter screening to pinpoint precise central points. Through functional connectivity and voxel‐wise seed point analysis, it is possible to more accurately observe functional changes in brain regions. This precision allows for a more targeted study of the mechanisms by which IMF improves motor functions (Wu et al. 2018; Metwali and Samii 2019).

### 4.3 IMF and Myelin

We observed myelin‐thickening changes and corresponding significant alterations in the expression levels of the proteins MBP and MAG. The MO and CC are core brain regions in motor‐related research. The MO contains multiple subregions (Lazari et al. 2024), such as anterior‐lateral motor cortex, anterior‐lateral and posterior‐medial, responsible for basic and advanced motor abilities (Lee, Kim, and Kaang 2022), respectively, and corresponding to similar components found in humans. The CC has long been studied as a center for balance control, with functions including interhemispheric communication, motor coordination, and emotional regulation (Edwards et al. 2020; Huang et al. 2023). Its contribution as a white matter component to rsfMRI findings has recently been validated. The increased expression of myelin‐related proteins such as MBP and MAG in the IMF group suggests that IMF promotes myelin formation and stability, which are essential for proper neuronal function (Huang et al. 2023). Enhanced myelination can improve the speed and efficiency of neuronal signaling, contributing to the observed improvements in motor functions. The findings of increased MBP and MAG expression are consistent with previous studies showing the beneficial effects of dietary interventions on myelination (Langley, Triplet, and Scarisbrick 2020).

Myelin formation is critically dependent on energy metabolism, as the unique composition and abundance of lipids in myelin, such as cholesterol and glycosphingolipids, are essential for its development, maintenance, and function. The dysregulation of these lipids has been linked to various neurological diseases (Barnes‐Vélez, Aksoy Yasar, and Hu 2023). Ex vivo studies have shown that oligodendrocytes can survive glucose deprivation by relying on fatty acid metabolism. These findings indicate that myelin integrity undergoes corresponding adjustments under different metabolic conditions (Asadollahi et al. 2024). These findings suggest that an imbalance between myelin synthesis and degradation may underlie myelin thinning observed in aging and disease. Our study has significant clinical implications for using IMF as a non‐pharmacological intervention at the systemic and organismal level to promote brain health and mitigate age‐related cognitive decline.

The observed improvements in physical, along with brain connectivity and enhanced myelination suggest that IMF may be a viable strategy to enhance the quality of life and prolong the independence of aging individuals. The potential of IMF to modulate key pathways involved in aging, such as oxidative stress, mitochondrial function, and neuroinflammation, further supports its use as a therapeutic approach for aging‐related conditions (Longo Valter and Mattson Mark 2014; Langley, Triplet, and Scarisbrick 2020).

## 5 Conclusions

In summary, our study demonstrated that IMF significantly positively impacted physical functions in aged mice. The findings of enhanced locomotor activity, motor coordination, and muscle strength, identified through rsfMRI multiparameter screening, highlighted MO and CC as key brain regions. Detailed analysis using TEM and IHC revealed significant changes in the expression levels of axon‐related proteins MBP and MAG, along with improved brain connectivity. These results underscore the potential of IMF as a dietary intervention to promote brain health and mitigate age‐related declines in motor and cognitive functions. The integration of behavioral assessments with advanced imaging techniques provides a comprehensive understanding of the effects of IMF on aging, offering valuable insights for future research and clinical applications.

## Author Contributions

Z.L., Z.Z., A.D., H.W., and J.W. design the experiments and wrote the manuscript. Z.L., Z.Z., Q.Z., and M.L. performed the whole experiments. Q.Z. and Z.G. performed the animal modeling. Z.L., Z.Z., H.D., J.W., Y.G., and A.D. perform the data analysis. H.D., A.D., H.W., and J.W. revised the manuscript. J.W., N.Z., and Y.G. provided funding and designed the project. All authors read and approved the manuscript.

## Conflicts of Interest

The authors declare no conflicts of interest.

## Supporting information


**Data S1.** Supporting Information.

## Data Availability

The data in this study are available from the corresponding author upon reasonable request.
