# Supplementary material for: Intermittent Fasting Enhances Motor Coordination Through Myelin Preservation in Aged Mice
Source: Aging Cell. 2025 Jan 8;24(5):e14476. doi: 10.1111/acel.14476 (PMC12074029; doi:10.1111/acel.14476)
Supplement: Supplementary file 1 — Data S1. Supporting Information. [file ACEL-24-e14476-s001.docx]

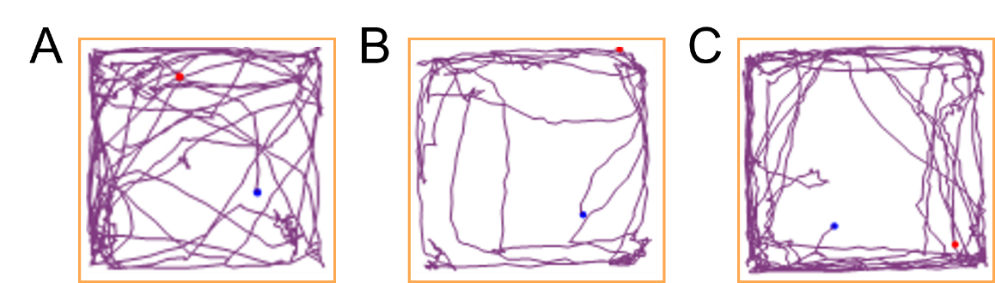


**Figure S1.** **The sample of tracking plot for open field test (OFT)**.

(A) Young control; (B) Old control; (C) IMF.


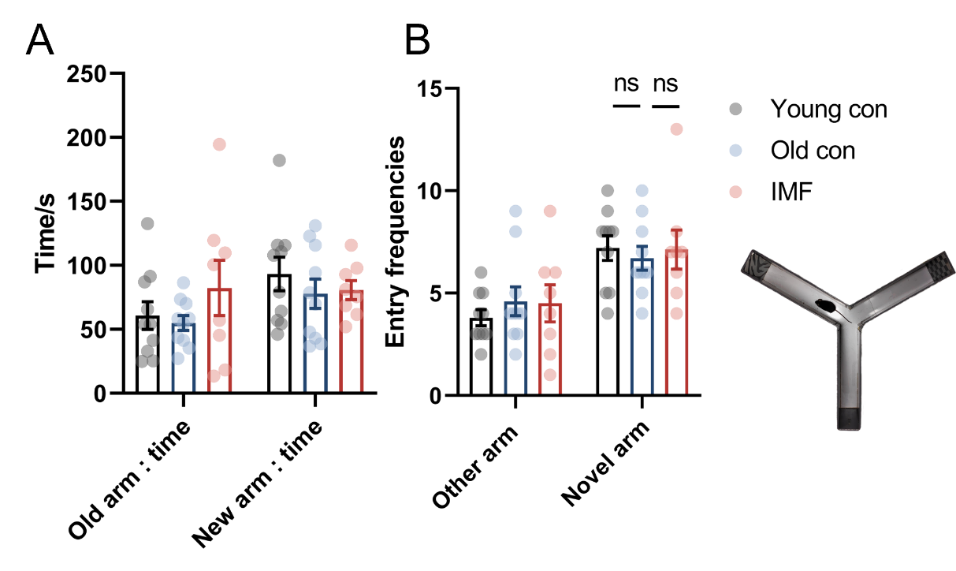


**Figure S2. Results of Y-maze test**.

(A) Time spent in new and old arms; (B) Frequency of entries into new and old arms.


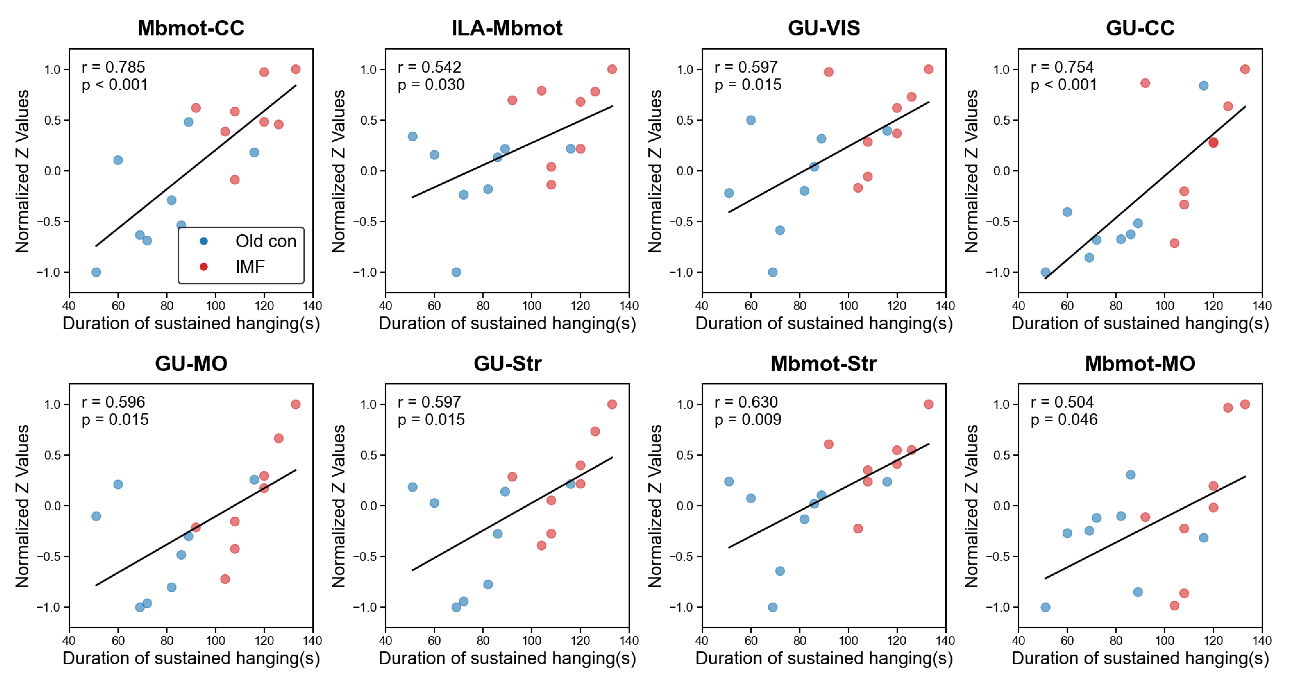


**Figure S3. Correlation analysis between Normalized Z Values of FC and Hanging time**

Significant correlations (Pearson’s r) between the Normailed Z Values of rsFC in the Mbmot-CC,ILA-Mbmot,GU-VIS,GU-CC,GU-MO,GU-Str,Mbmot-Str,Mbmot-MO pathways and the Hanging time in HT. P-values were corrected with FDR. The blue dots represent the data of OLD con mice (n = 8). The red dots represent the data of IMF mice (n =8).


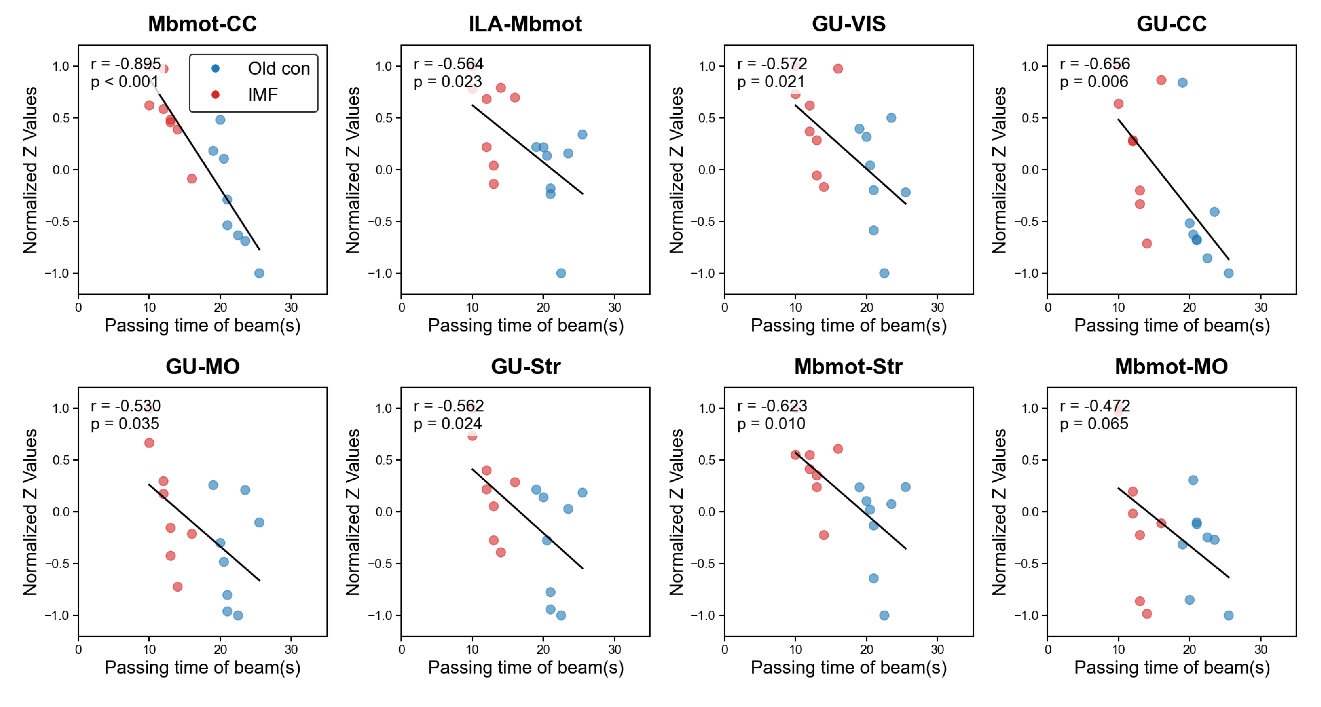


**Figure S4. Correlation analysis between Normalized Z Values of FC and Passing time**

Significant correlations (Pearson’s r) between the Normailed Z Values of rsFC in the Mbmot-CC,ILA-Mbmot,GU-VIS,GU-CC,GU-MO,GU-Str,Mbmot-Str,Mbmot-MO pathways and the Passing time in BT. P-values were corrected with FDR. The blue dots represent the data of OLD con mice (n = 8). The red dots represent the data of IMF mice (n =8).

**Table S1. Abbreviations and corresponding full terms of the research of interest (ROI) in the Allen standard partition.**

| **Label** | **Abbr** | **sub- regions** | **roi** |
| --- | --- | --- | --- |
| 1 | FRP | Frontal pole cerebral cortex | Isocortex |
| 2 | MO | Somatomotor areas | Isocortex |
| 3 | SS | Somatosensory areas | Isocortex |
| 4 | SSs | Supplemental somatosensory area | Isocortex |
| 5 | GU | Gustatory areas | Isocortex |
| 6 | VISC | Visceral area | Isocortex |
| 7 | AUD | Auditory areas | Isocortex |
| 8 | VIS | Visual areas | Isocortex |
| 9 | ACA | Anterior cingulate area | Isocortex |
| 10 | PL | Prelimbic area | Isocortex |
| 11 | ILA | Infralimbic area | Isocortex |
| 12 | ORB | Orbital area | Isocortex |
| 13 | AI | Agranular insular area | Isocortex |
| 14 | RSP | Retrosplenial area | Isocortex |
| 15 | PTLp | Posterior parietal association areas | Isocortex |
| 16 | Tea | Temporal association areas | Isocortex |
| 17 | PERI | Perirhinal area | Isocortex |
| 18 | ECT | Ectorhinal area | Isocortex |
| 20 | HPF | Hippocampal formation | HPF |
| 21 | CTXsp1 | Cortical subplate | CTXsp |
| 22 | CA3 | Striatum | HPF |
| 23 | RHP | Retrohippocampal region | HPF |
| 24 | CTXsp2 | Cortical subplate | CTXsp |
| 25 | Str | Striatum | CPU |
| 26 | PALm | Pallidum medial region | CPU |
| 27 | VENT | Ventral group of the dorsal thalamus | TH |
| 28 | DORpm | Thalamus polymodal association cortex related | TH |
| 29 | MEZ | Hypothalamic medial zone | HY |
| 30 | Mbmot | Midbrain motor related | MB |
| 31 | P-mot | Pons motor related | HB |
| 32 | CC | Corpus callosum | CC |

**Table S2. Collection of the statistical analysis of significant different functional connectivity analysis for the pairs of brain regions.**

| Brain regions | p | t |
| --- | --- | --- |
| MO-ACA | 0.0100 | 2.8989 |
| GU-VIS | 0.0096 | 2.9194 |
| AUD-CA3 | 0.0024 | 2.9399 |
| AUD-MEZ | 0.0057 | 2.9604 |
| VIS-Str | 0.0007 | 2.9809 |
| VIS-CC | 0.0008 | 2.1001 |
| ACA-PTLp | 0.0007 | 2.1022 |
| ILA-Mbmot | 0.0054 | 2.1042 |
| PTLp-CC | 0.0002 | 2.1063 |
| Tea-CA3 | 0.0026 | 2.1083 |
| ECT-HPF | 0.0098 | 2.1104 |
| ECT-CTXsp1 | 0.0100 | 2.1124 |
| ECT-CA3 | 0.0006 | 2.1145 |
| HPF-CA3 | 0.0093 | 2.1165 |
| CTXsp1-CA3 | 0.0062 | 2.1186 |
| CA3-VENT | 0.0053 | 2.1206 |
| DORpm-MEZ | 0.0055 | 2.1227 |

**Table S3. Detailed information for NMI values for each subregion.**

| No | Region | NMI |
| --- | --- | --- |
| 1 | FRP | 0.4602 |
| 2 | MO | 0.4494 |
| 3 | SS | 0.5949 |
| 4 | SSs | 0.7144 |
| 5 | GU | 0.5204 |
| 6 | VISC | 0.3807 |
| 7 | AUD | 0.4621 |
| 8 | VIS | 0.4385 |
| 9 | ACA | 0.3316 |
| 10 | PL | 0.3843 |
| 11 | ILA | 0.4060 |
| 12 | ORB | 0.4582 |
| 13 | AI | 0.5574 |
| 14 | RSP | 0.5301 |
| 15 | PTLp | 0.4063 |
| 16 | Tea | 0.4505 |
| 17 | PERI | 0.5423 |
| 18 | ECT | 0.5352 |
| 19 | HPF | 0.4482 |
| 20 | CTXsp1 | 0.3730 |
| 21 | CA3 | 0.3745 |
| 22 | RHP | 0.4135 |
| 23 | CTXsp2 | 0.4179 |
| 24 | Str | 0.4429 |
| 25 | PALm | 0.4438 |
| 26 | VENT | 0.4332 |
| 27 | DORpm | 0.4709 |
| 28 | MEZ | 0.5530 |
| 29 | Mbmot | 0.5571 |
| 30 | P-mot | 0.4809 |
| 31 | CC | 0.4553 |
